# Profiling BRCA1-BRCT interactions and their functional relevance at amino acid resolution

**DOI:** 10.1093/nar/gkaf848

**Published:** 2025-09-17

**Authors:** Venda Mangkusaputra, Andrea G Murachelli, Zhengzhou Yu, Annouche den Hollander, Roberta Menafra, Anne Schreuder, Susan L Kloet, Titia K Sixma, Sylvie M Noordermeer

**Affiliations:** Department of Human Genetics, Leiden University Medical Center (LUMC), Einthovenweg 20, 2333 ZC Leiden, The Netherlands; Oncode Institute, Jaarbeursplein 6, 3521 AL Utrecht, The Netherlands; Oncode Institute, Jaarbeursplein 6, 3521 AL Utrecht, The Netherlands; Netherlands Cancer Institute (NKI), Plesmanlaan 121, 1066 CX Amsterdam, The Netherlands; Department of Human Genetics, Leiden University Medical Center (LUMC), Einthovenweg 20, 2333 ZC Leiden, The Netherlands; Department of Human Genetics, Leiden University Medical Center (LUMC), Einthovenweg 20, 2333 ZC Leiden, The Netherlands; Leiden Genome Technology Center, LUMC, Einthovenweg 20, 2333 ZC Leiden, The Netherlands; Department of Human Genetics, Leiden University Medical Center (LUMC), Einthovenweg 20, 2333 ZC Leiden, The Netherlands; Oncode Institute, Jaarbeursplein 6, 3521 AL Utrecht, The Netherlands; Leiden Genome Technology Center, LUMC, Einthovenweg 20, 2333 ZC Leiden, The Netherlands; Oncode Institute, Jaarbeursplein 6, 3521 AL Utrecht, The Netherlands; Netherlands Cancer Institute (NKI), Plesmanlaan 121, 1066 CX Amsterdam, The Netherlands; Department of Human Genetics, Leiden University Medical Center (LUMC), Einthovenweg 20, 2333 ZC Leiden, The Netherlands; Oncode Institute, Jaarbeursplein 6, 3521 AL Utrecht, The Netherlands

## Abstract

BRCA1 (breast cancer-associated protein 1) plays a central role in homologous recombination (HR) through interactions with multiple proteins across its various domains. The C-terminal BRCT domains bind HR regulators such as ABRAXAS1, CtIP, and BRIP1, each contributing to distinct, sometimes opposing, functions. While pathogenic mutations frequently cluster within the canonical BRCT phospho-binding pocket, the broader mutational landscape and its functional consequences remain poorly understood. Here, we used a site-saturation mutagenesis library of the BRCT domains to test >4,000 single-residue variants for their ability to bind ABRAXAS1 and CtIP. Using a yeast two-hybrid screen, we systematically assessed these interactions and validated key findings in mammalian cells. The resulting interaction map identified previously uncharacterized residues critical for partner binding and demonstrated their detrimental impact on HR. Importantly, we identified separation-of-function mutations that selectively disrupt individual protein interactions, enabling a more detailed analysis of each partner’s contribution to HR. Functional assays suggested that disruption of CtIP binding had the most pronounced impact on HR. Furthermore, integration of our data with clinical variant data revealed a strong correlation between loss of protein binding and pathogenicity, highlighting the potential utility of our interaction map for clinical variant interpretation.

## Graphical Abstract

**Graphical Abstract:**
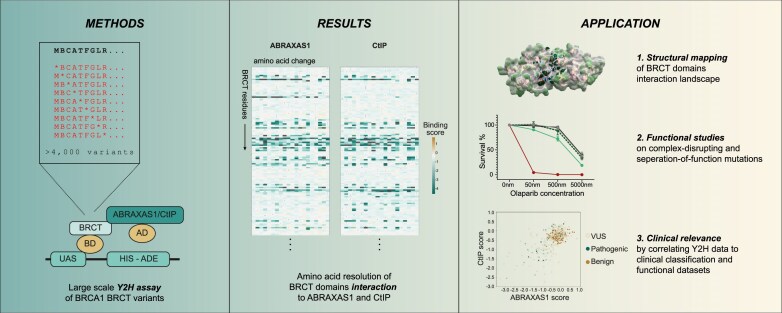

## Introduction

BRCA1 (breast cancer-associated protein 1) is a critical tumour suppressor strongly linked to the development of several cancers, particularly hereditary breast and ovarian cancer [1, 2]. Its tumour suppressor function has traditionally been attributed to its essential role in homologous recombination (HR), a high-fidelity DNA double-strand break (DSB) repair pathway crucial for maintaining genome integrity [3–7]. Recent studies have expanded this understanding, demonstrating that BRCA1 deficiency not only impairs HR but also leads to the accumulation of genomic instability due to its role in replication fork protection and the suppression of single-stranded DNA (ssDNA) gaps [8–14]. The inability of BRCA1-deficient cells to effectively repair these lesions further exacerbates genomic instability, ultimately defining the pathogenic consequences of BRCA1 mutations [15].

**Figure 1.**
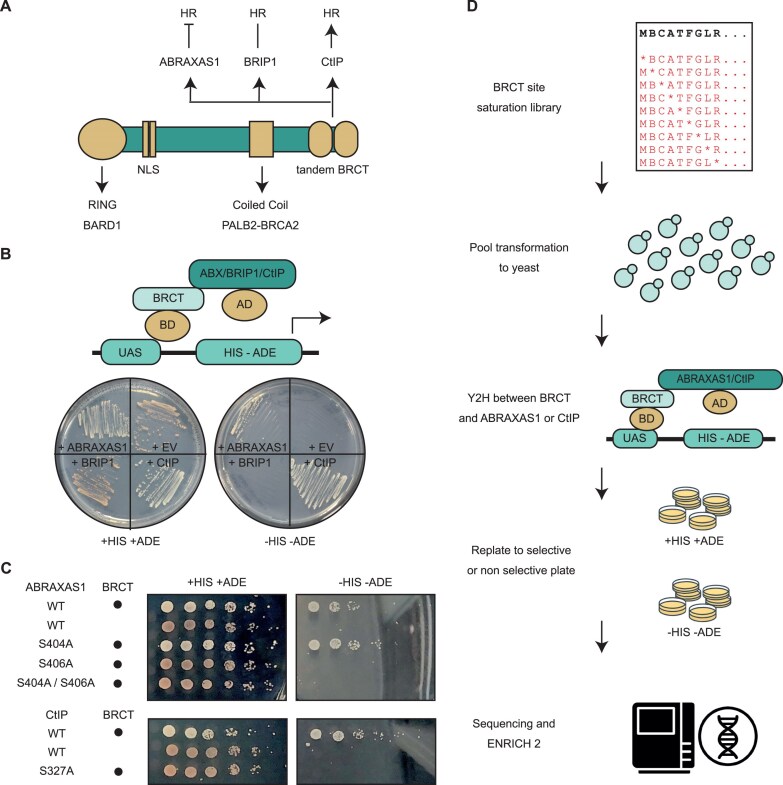
Yeast two-hybrid (Y2H) setup to evaluate BRCT binding characteristics. (**A**) Schematic representation of BRCA1 protein domains and their key interacting partners. (**B**) Top: schematic representation of Y2H setup. Bottom: restreaking assay of yeast colonies on non-selective medium [+histidine and adenine (+HIS +ADE)] or selective medium [−histidine and adenine (−HIS −ADE)], demonstrating binding interactions between BRCT fused to the DNA-binding domain (BD; present in all conditions) and ABRAXAS1 or CtIP fused to the activation domain (AD). EV stands for empty vector. (**C**) Yeast drop dilution assay of yeast cultures spotted on non-selective medium (+HIS +ADE) or selective medium (−HIS −ADE), showing that the interaction of BRCT domains with ABRAXAS1 and CtIP depends on S406 and S327, respectively. (**D**) Schematic overview of the large-scale Y2H workflow.

In the process of HR, BRCA1 functions at multiple stages. It promotes DNA end resection, a key step that channels repair towards HR instead of the more error-prone non-homologous end joining (NHEJ) [16–18]. Additionally, BRCA1 facilitates the recruitment and loading of RAD51, a critical factor in the downstream step of strand invasion and exchange [19–21]. Given that BRCA1 is a multi-domain protein interacting with numerous partners, its function in HR is highly dependent on its interaction network (Fig. 1A) [22].

The tandem BRCT (BRCA1 C-terminal) domains, located at the C-terminus of BRCA1, are particularly intriguing due to their role in mediating phospho-dependent interactions with multiple proteins, including ABRAXAS1, BRIP1, and CtIP [23–26]. A common phosphorylated motif in these three proteins competes for a shared binding pocket within the BRCT domains [27]. Notably, BRCA1’s function in HR varies depending on which protein is bound. Previous studies have indicated that disrupting binding between CtIP and BRCA1 reduces HR efficiency [16, 18, 28], whereas ABRAXAS1 absence may enhance HR levels [29, 30]. The role of BRIP1 in HR remains open for discussion, with conflicting reports regarding its function [9, 23, 31–33].

The crystal structure of the BRCT domains bound to phospho-peptides of partner proteins has been resolved. This provides valuable insight into a conserved phospho-binding pocket involving core BRCT residues 1655, 1656, 1699, 1701, 1702, 1704, 1775, and 1839 [34–39]. Many pathogenic missense mutations cluster within this pocket and are known to impair HR, indicating the essential role of this domain for BRCA1 function. For instance, mutations at arginine 1699, such as R1699W, result in HR deficiency and are clinically recognized as pathogenic [40]. Outside this well-characterized binding pocket, other mutations within the BRCT domains have also been associated with HR defects and/or pathogenicity [41–43]. However, whether their effects are due to disrupted protein interactions remains unclear. Additionally, certain mutations may selectively affect interactions with individual partner proteins, but their precise effect on HR and cancer development is not well understood.

The available crystal structures of the BRCT domains binding their interactors involve only a minimal 11- or 12-amino-acid-long phospho-peptide from ABRAXAS1, BRIP1, or CtIP [34–39] including the core motif of pS-x-x-F [25]. We performed structural predictions for the BRCT-mediated interactions with the full-length proteins using AlphaFold3, which accurately recapitulated the known interactions and hinted at a more extensive interface beyond what is resolved in the crystal structures. However, these additional regions were predicted with low confidence, indicating a high degree of uncertainty in their contribution to binding. Hence, a detailed characterization of BRCT domain interactions with partner proteins is critical for elucidating their functional roles and to understand the consequences of specific mutations.

To further explore the BRCT-driven interactions, we systematically investigated the BRCT domains by generating a site-saturated mutagenesis library of this domain, resulting in over 4,000 mutants. We then assessed the binding of these mutants to ABRAXAS1 and CtIP using a Y2H assay, which enabled the construction of an extensive interaction landscape of the BRCT domains with their partner proteins. Multiple validation experiments in mammalian cells demonstrated the accuracy of this interaction map, reinforcing its relevance in a physiological context.

Using our data, we identified previously uncharacterized residues in the BRCT domains that are critical for interactions with ABRAXAS1, BRIP1, and CtIP, and we demonstrated their detrimental effects on HR. Furthermore, we identified specific mutations that differentially affected binding to the three partner proteins, with functional experiments showing varying degrees of HR impairment. Notably, the loss of CtIP binding resulted in the most pronounced impact on HR.

In a broader context, we conducted a comparative analysis with previously published large-scale mutational datasets [41, 42]. This analysis confirmed a strong correlation between the loss of BRCT protein binding and pathogenicity, underscoring the potential utility of our data in refining the classification of BRCA1 mutations. Furthermore, the analysis suggested that the role of the BRCT domains in HR extends beyond its interactions with ABRAXAS1, BRIP1, and CtIP, pointing to additional, yet under-explored mechanisms of BRCA1-mediated HR regulation.

## Materials and methods

### Molecular biology techniques

Polymerase chain reaction (PCR) for cloning purposes was performed using Phusion High-Fidelity DNA Polymerase (Thermo Fisher Scientific, Waltham, MA, USA) according to the manufacturer’s instruction. Diagnostic PCR was performed using Kappa HiFi (Roche, Basel, Switzerland) according to the manufacturer’s instruction. All genomic DNA (gDNA) and plasmid isolation, or PCR fragment purification was performed using commercially available kits (Macherey Nagel, Düren, Germany) according to the manufacturer’s instruction.

Triple TY1-tagged BRCA1 mutations were introduced into pCW57.1 plasmids using site-directed mutagenesis. Two separate PCR reactions were performed, each containing one primer with the desired mutation and another primer annealing to the plasmid backbone (Supplementary Table S1). The resulting PCR products were assembled into the intact plasmid using Gibson assembly (NEB, Ipswich, MA, USA) following the manufacturer’s instructions.

For *Escherichia coli* transformation, DH5α competent cells were transformed by heat shock at 42°C for 45 s. Yeast transformation was performed based on the LiAc/ssDNA/PEG method [44].

### Yeast cell culture

All *Saccharomyces cerevisiae* yeast strains used were derived from PJ69-4A (MATa gal4 gal80 his3-delta200 leu2-3 leu2-112 trp1-delta901 LYS2::GAL1-HIS3 ade2::GAL2-ADE2 met2::GAL7-lacZ), kindly gifted by Paul van Heusden (Leiden University, The Netherlands). Yeast strains were cultivated at 30°C in YPD consisting 10 g/l Bacto Yeast extract, 20 g/l Bacto Peptone, and 20 g/l glucose. Yeast strains containing plasmids were cultivated at 30°C in SC (synthetic complete) drop out media (MP Biomedicals, Irvine, CA, USA) supplemented with 5 g/l ammonium sulphate, 1.7 g/l yeast nitrogen base, and 20 g/l glucose. YPD and SC pH were calibrated to 5.8 with NaOH. Agar YPD or SC was made by adding 20 g/l agar.

### Mammalian cell line and cell culture

RPE1 hTERT cells were obtained from ATCC (Manassas, VA, USA). RPE1 hTERT *PAC*^−/−^*TP53*^−/−^ and RPE1 hTERT *PAC*^−/−^*TP53*^−/−^*BRCA1*^−/−^ (referred to as *BRCA1 KO* in this manuscript) cells were created by nucleofection of pLentiCRISPR_v2 with the following sgRNAs: sg*PAC*: ACGCGCGTCGGGCTCGACAT; sg*TP53*: CAGAATGCAAGAAGCCCAGA; sg*BRCA1*: AAGGGTAGCTGTTAGAAGGC. Single clones were expanded and confirmed for knock-out by performing PCR amplification at the targeted locus, followed by Sanger sequencing and TIDE analysis.

The *BRCA1* gene in RPE1 hTERT *PAC^−/−^ TP53^−/−^* cells was endogenously tagged at the C-terminus with a triple Ty1 tag (referred to as *TP53 KO* in this manuscript) by electroporation of the pLentiCRISPR-v2 vector with a *BRCA1* sgRNA: 5′-TGGCTGGCTGCAGTCAGTAG-3′ and a C-terminal tagging vector BRCA1-XhoI-tag-P2A-Neo with 500-bp homology arms annealing to BRCA1’s C-terminus, a Ty1(3x)-tag, P2A, and a neomycin resistance cassette derived from the previously described YFP-P2A-Neo vector [45]. Upon neomycin selection, single clones were expanded and confirmed by performing PCR amplification at the targeted locus, followed by Sanger sequencing.

*TP53 KO* cells were transfected using Lipofectamine LTX (Invitrogen) with pSpCas9(BB)2A_puro (PX459) containing the following guide sequence: 5′-CCAGCACCTCAACACGGACT-3′ to target *ABRAXAS1*. Seventy-two hours after transfection, cells were selected using 2 μg/ml puromycin for 48 h. Cells were allowed to recover for 48 h before they were seeded as single clones into 96-well plates. Single clones were screened by western blot and genomic PCR, followed by TIDE analysis.

RPE1 hTERT BRIP1 knock-out cells were described before [9].

The *CtIP* gene in *TP53 KO* were endogenously mutated at serine 327 to alanine. For this, crRNA (5′-CTAGAGGTAGCTCCAAATAC-3′) was mixed with tracrRNA in equimolar ratios, heated to 95°C, and cooled to room temperature to form a 50 μM sgRNA duplex. The sgRNA (150 pmol) was combined with 125 pmol Alt-R Cas9 enzyme (IDT, Newark, NJ, USA) and incubated at room temperature for 10–20 min to assemble the RNP complex. The P3 Primary Cell 4D-Nucleofector X Kit (Lonza Bioscience, Basel, Switzerland) was then used according to the manufacturer’s protocol to deliver the RNP complex and Ultramer ssODN donor templates (*CtIP*: 5′-TTTTCAGATTCTACTTCAAAGACTCCTCCTCAAGAAGAATTACCTACTCGAGTGTCAGCTCCAGTGTTCGGTGCCACATCCAGTATCAAAAGTGGTTTAGATTTGAATACAAGTTTGTCCCCTTCTCTTTTACAGCCTGGGAA-3′). Electroporation was performed using the Amaxa 4D Nucleofector X-Unit (Lonza Bioscience) with program EA-104. Single clones were expanded and confirmed by performing PCR amplification at the targeted locus, followed by Sanger sequencing.

BRCA1 wild-type (WT) or the different BRCA1 mutants were reintroduced to *BRCA1 KO* cells by lentiviral transduction of pCW57.1 plasmid containing triple TY1-tagged BRCA1 WT [46] or mutants (referred to as WT or BRCA1 mutants in this manuscript).

HEK 293T cells were obtained from ATCC (Manassas). RPE1 and HEK 293T were cultured in Dulbecco’s modified Eagle’s medium (DMEM) high glucose, GlutaMAX, and pyruvate supplemented (Thermo Fisher Scientific) + 10% fetal calf serum (FCS) and 1% penicillin + streptomycin (Pen-Strep).

### Yeast two-hybrid assay

Human BRCA1 BRCT domains (aa 1646–1859 of the human NP_009225), human ABRAXAS1, human BRIP1, or human CtIP was cloned from the addgene plasmids #27495, #17642, and #71109 into plasmid pGBKT7 DNA-BD (cat #630443, Takara Bio, Kusatsu, Japan) or pGADT7 AD (cat #630442, Takara Bio) kindly gifted by Paul van Heusden to fuse them into DNA binding domain (BD), or activation domain (AD), respectively. pGBKT7 and pGADT7 fused to partner proteins were co-transformed into *S. cerevisiae* PJ69-4A and plated on agar plates containing SC lacking leucine and tryptophan (SC-LEU-TRP). Single colonies were picked and re-streaked into SC-LEU-TRP and SC lacking leucine, tryptophan, histidine, and adenine (SC-LEU-TRP-HIS-ADE) to visualize binding.

### Yeast drop test

Yeast culture was grown overnight, followed by dilution in sterile milliQ to OD of 0.5. Six rounds of 5× serial dilution were made and 4 μl of each dilution was spotted on YPD/SC agar plates.

### Large-scale yeast two-hybrid assay

An amino acid site-saturation library pool of BRCT variants (BRCA1 residues 1646–1859) was ordered from Twist Bioscience (San Francisco, CA, USA), and cloned into pGBKT7 DNA-BD plasmid by Gibson assembly. Ten Gibson assembly reactions with 1:5 molar ratio of backbone (50 ng) to insert (31 ng) were performed to aim for 70x library coverage. Each reaction was transformed into 150 μl of DH5α competent cells and grown overnight in total 500 ml LB medium + ampicillin. The plasmid pool was isolated and the presence of all variants in the pool was confirmed by Illumina sequencing on an Illumina MiSeq. Due to the 250 bp read length limitation of Illumina sequencing, the BRCT domains were sequenced in three overlapping fragments. Amplification of fragments was done with primer pairs listed in Supplementary Table S1. To address the inability to determine whether a fragment represents the true WT BRCT or whether it carriers a mutation located in another fragment, a WT plasmid containing synonymous mutations every 15 amino acids was spiked in. This plasmid served as a true WT reference. pGBKT7 DNA-BD containing a pre-mature stop codon directly after the binding domain sequence was also spiked in as a negative control for binding.

Five micrograms of plasmid pool was transformed into PJ69-4A expressing pGADT7 AD fused to ABRAXAS1 or CtIP and plated onto 50 x 15 cm agar plates containing SC-LEU-TRP for selection to keep ∼100x library coverage. All colonies were scraped and pooled, and then replated onto 25 × 15 cm agar plates containing SC-LEU-TRP and 25 × 15 cm agar plates containing SC-LEU-TRP-HIS-ADE to keep around 50x library coverage. Colonies from the SC-LEU-TRP plates were pooled separately from those on the SC-LEU-TRP-HIS-ADE plates. Replating of pooled transformants was done in three replicates. Total yeast DNA, including plasmid DNA, was isolated using a Yeast DNA Extraction Kit (Thermo Fisher Scientific) and 100 ng was used as input for targeted amplification of the BRCT domains. PCR reactions were performed using 2× Kapa HiFi master mix (Roche, KR0370) using the following conditions: 98°C for 1 min; 18 cycles of 98°C for 20 s, 65°C for 30 s, and 72°C for 30 s; and 72°C for 2 min. After clean-up with Ampure XP beads (Beckman Coulter) the PCR product was checked on a Agilent BioAnalyzer 2100 HS chip. A second PCR with Illumina index primers was performed under the following conditions: 98°C for 1 min; 10 cycles of 98°C for 20 s, 60°C for 30 s, and 72°C for 30 s; and 72°C for 2 min. The resulting PCR products were equimolarly pooled. All samples were sequenced on an Illumina MiSeq.

Fastq files were pre-processed to remove the primers sequences using cutadapt v 4.0. ENRICH2 [47] v1.3.1 was used to determine depletion score of each mutant, based on the sequencing read counts. We used the score of the overlapping amino acids (3–4 amino acids) to normalize the score between the three BRCT fragments. ENRICH2 does not calculate a score for mutants undetected (absent) in the NGS data of the yeast pool grown in selection medium. These mutants were marked as N/A or #VALUE in Supplementary Table S2 that provides the ENRICH2 output. For further analysis and visualisation purposes, we manually curated these mutants with a score of −5 in the subsequent figures and Supplementary Table S3.

### Viral transductions and transfection

Lentivirus production was carried out in HEK 293T cells using jetPEI transfection reagent (Polyplus Transfection, Illkirch, France). Cells were transfected with the pCW57.1 plasmid, supplemented by third-generation packaging vectors pMDLg/pRRE, pRSV-Rev, and pMD2.G. Viral supernatants were collected 48–72 h post-transfection, filtered using a 0.45-μm filter, and applied to target cells at a multiplicity of infection (MOI) of 1 in the presence of 4 μg/ml polybrene. For transduction of RPE1 *PAC*^−/−^ cells, 2 μg/ml puromycin was used for selection.

For transient expression of BRCA1 WT or mutants in HEK 293T, pCW57.1 plasmids containing BRCA1 WT/mutants were transfected using jetPEI transfection reagent. Expression of BRCA1 constructs was induced with 2 μg/ml doxycycline.

### Western blotting

RIPA lysis buffer (1% NP-40, 50 mM Tris–HCl, pH 7.5, 150 mM NaCl, 0.1% SDS, 3 mM MgCl_2_, 0.5% sodium deoxycholate) supplemented with 100 U/ml Benzonase (Sigma–Aldrich) and Complete Protease Inhibitor Cocktail (Sigma–Aldrich) was used for cell lysis. LDS or sodium dodecyl sulphate sample buffer with dithiothreitol (DTT) was added to the lysates, followed by denaturation at 95°C for 5 min. Proteins were separated by sodium dodecyl sulphate–polyacrylamide gel electrophoresis on 4%–12% gradient gels (Thermo Fisher Scientific) and transferred to Amersham Protran premium 0.45-μm nitrocellulose membranes (GE Healthcare Life Sciences, Chicago, IL).

Membranes were blocked in 8% skimmed milk (Santa Cruz Biotechnology, Dallas, TX) in 1× PBS + 0.05% Tween or in blocking buffer for fluorescent western blotting (Rockland, Pottstown, PA, USA). Primary and secondary antibody incubations were performed in respective blocking buffer. Membranes were treated with the WesternBright ECL HRP Substrate Kit (Advansta, San Jose, CA, USA) and imaged using an Amersham Imager 680 (Bioke, Leiden, The Netherlands) or New Oddyssey (LI-COR Biosciences, Lincoln, Nebraska, USA).

The following primary antibodies were used: TY (Diagenode, C15200054), BRCA1 (Millipore, 07-434), ABRAXAS1 (Abcam, ab139191), BRIP1 (Sigma–Aldrich, B1310-200), CtIP (Millipore, MABE1060), BARD1 (Bethyl, A300-263A), PALB2 (Cell Signaling Technology, #30253), and Tubulin [Sigma–Aldrich, T6199 (Clone DM1A)].

### Colony formation assay

RPE1 cells were seeded onto 10-cm dishes. Five hundred cells were seeded for T*P53 KO*, ABRAXAS1 KO, *BRIP KO, CtIP-S327A* or cells re-expressing BRCA1 WT, or BRCA1 mutants, while 1500 cells were seeded for *BRCA1 KO* cells. Cells were treated in 10 ml of medium containing 0, 50, 500, or 5000 nM olaparib. Medium was supplemented with 2 μg/ml dox for expression of BRCA1 WT or mutants. Medium was refreshed after 7 days. Colonies were stained with crystal violet (0.4% w/v) after 14 days and counted manually.

### IR-induced foci immunofluorescence

Cells were seeded on sterile 13-mm glass coverslips. Twenty-four hours upon seeding, 2 μg/μl of doxycycline was supplemented to the medium. Cells were grown for 24 h until 85% confluency and pre-extracted with ice cold nuclear extraction (NuEx) buffer (20 mM HEPES, pH 7.5, 20 mM NaCl, 5 mM MgCl_2_, 1 mM DTT, 0.5% NP-40 (IGEPAL CA-630, Sigma–Aldrich), 1× Complete Protease Inhibitor Cocktail (Sigma–Aldrich) for 12 min at 4°C, followed by fixation with 2% (w/v) paraformaldehyde in PBS for 20 min at room temperature at 1, 3, or 6 h upon irradiation. Cells were subsequently incubated in primary antibody in PBS+ ((5 g/L BSA (Sigma Aldrich) and 1.5 g/L glycine (Sigma Aldrich) in PBS) for 1.5–2 h, washed 5× with PBS, and incubated with secondary antibody and DAPI in PBS+ for 1 h. Cells were washed 5× with PBS and mounted with Aqua-Poly/mount (Poly sciences, Warrington, PA, USA). Pictures were made of at least 100 cells per condition per replicate using the Zeiss Axio Imager 2 fluorescent microscope at a 68× zoom. Foci of at least 100 cells per condition per replicate were quantified using the IRIF analysis 3.2 Plugin in ImageJ.

### BRCT purification

A single colony of *E. coli* BL21 containing HIS-BRCT construct plasmid was inoculated into 20 ml LB medium supplemented with kanamycin (LB + Kan) and grown overnight at 37°C with shaking at 220 rpm. The overnight culture (2.5 ml) was inoculated into 1 l of LB + Kan and grown at 37°C with shaking at 200 rpm until the OD_600_ reached 0.6. A 200 μl sample was taken as a control. Induction of the rest of the culture was performed by adding 1 ml of 100 mM isopropyl β-D-1-thiogalactopyranoside (IPTG), and the culture was grown overnight at 18°C with shaking at 200 rpm.

Cultures were centrifuged for 15 min at 6000 × *g* at 4°C, and the bacterial pellet was resuspended in 40 ml of bacterial lysis buffer [300 mM NaCl, 20 mM HEPES, pH 7.5, 1× AEBSF, protease inhibitor (1 tablet/50 ml)]. The resuspended cells were sonicated three times at 50% amplitude for a total of 3 min (15 s on, 30 s off) in a narrow 100 ml beaker placed in ice. Following sonication, 2.1 ml lysozyme (10 mg/ml), 98 μl benzonase (25 U/μl), and 80 μl MgCl_2_ (1 M) were added to the lysate, and the mixture was incubated for 30 min at 4°C with rotation. The lysate was centrifuged for 40 min at 18 000 × *g* at 4°C, and the supernatant was filtered through a 0.45 μm filter. A 50 μl sample of the lysate was taken as a control.

Nickel-NTA agarose beads (2 ml) were washed three times with 10 ml wash buffer (300 mM NaCl, 20 mM HEPES, pH 7.5, 20 mM imidazole) and added to the filtered lysate. The mixture was incubated for 2 h at 4°C with rotation. The lysate-bead mixture was applied to a column, and the flow-through was collected. The beads were washed with bacterial lysis buffer until the flow-through tested negative for protein by Bradford assay. A 50 μl flow-through sample was collected for control. The column was closed, and 3 ml of elution buffer (300 mM NaCl, 20 mM HEPES, pH 7.5, 300 mM imidazole) was added to the beads, followed by a 15-min incubation at room temperature. The eluted protein was collected as the flow-through. The beads were washed three times with 1 ml of elution buffer, and the flow-through was tested using the Bradford assay until negative. Fifty microlitre samples were taken for control.

The eluted protein was dialyzed at 4°C in 4 l of dialysis buffer (300 mM NaCl, 20 mM HEPES, pH 7.5, 10 mM imidazole, 1 mM DTT) for 1.5 h, followed by overnight dialysis in fresh 4 l of dialysis buffer. Protein concentration was measured following dialysis.

### Yeast pull-down assay

A 200 ml yeast culture was grown from an OD_600_ of 0.2 to 1.0. Cells were pelleted by centrifugation and washed with 20 ml of 20 mM HEPES–KOH, pH 7.9, followed by 10 ml of lysis buffer (110 mM HEPES–KOH, pH 7.9, 50 mM potassium acetate, 10 mM magnesium acetate, 2 mM EDTA). The pellet was resuspended in an equal volume (w/v) of lysis buffer supplemented with 1× Complete Protease Inhibitor Cocktail (Sigma–Aldrich), 1 M DTT, 1 M E64, phosphatase inhibitor (Roche), and 1% Tricylol. The resuspended cells were frozen in liquid nitrogen and ground into a powder. The powder was weighed, and 2.5 g was used per immunoprecipitation (IP) experiment. The powder was dissolved in 3 ml of glycerol mix [11.4% (v/v) lysis buffer, 50% (v/v) glycerol, 200 mM potassium acetate, 0.5% (v/v) IGEPAL, 1 M DTT, and 1× Complete Protease Inhibitor Cocktail] and incubated overnight.

HIS nickel affinity gel beads (Sigma–Aldrich) were used for the pull-down assays. Beads (100 μl/reaction) were blocked in wash buffer (100 mM HEPES–KOH, pH 7.9, 110 mM potassium acetate, 10 mM magnesium acetate, 2 mM EDTA, 0.1% IGEPAL, 20 mM imidazole) for 30 min. The blocked beads (100 μl) were coupled to 10 μg of purified HIS-BRCT for 1 h in a total volume of 200 μl wash buffer. The beads were washed four times with 1 ml of wash buffer and resuspended in 400 μl of the same buffer. Benzonase (128 μl, 25 U/μl) was added to the dissolved yeast powder and incubated for 30 min with rocking. The sample was centrifuged at 10 000 × *g* for 30 min at 4°C. A 50 μl aliquot was taken as the input sample. Lysates (400 μl) were added to 400 μl of resuspended beads and incubated for 3 h at 4°C. Beads were washed four times with wash buffer, with 5-min incubations at each step. Finally, beads were eluted in 50 μl LDS/reduction buffer and boiled for 5 min. Samples were run on western blot.

### Mammalian cell immunoprecipitation

Twenty microlitres per sample of Pierce protein G magnetic beads (Thermo Fischer) were pre-washed three times with 1 ml of NETT buffer (100 mM NaCl, 50 mM Tris, pH 7.6, 5 mM EDTA, pH 8.0, 0.5% Triton, 1× Complete Protease Inhibitor Cocktail, 7 mM MgCl_2_) and resuspended in 20 μl NETT buffer. TY-antibody (2 μl/sample) was added to the beads, and the mixture was incubated for 2 h and 30 min at 11 rpm in a cold room. The beads were washed twice in NETT buffer and resuspended in 20 μl NETT buffer per sample.

Cells from a full T75 flask were washed once with 10 ml PBS, harvested, and lysed in 1 ml NETT buffer supplemented with 500 U benzonase. Lysis was performed for 45 min at 4°C with rotation. Lysates were centrifuged at 14 000 rpm for 10 min at 4°C. The supernatant was collected, and 40 μl was taken as a whole-cell extract (WCE) control. The remaining supernatant was used for IP.

Pretreated G beads (20 μl/sample) with bound TY-antibody were added to the lysates, and the mixture was rotated for 4 h at 4°C. Beads were washed five times with 1 ml NETT buffer, and the buffer was removed. The beads were resuspended in 20 μl of 2× LDS/reducing buffer and boiled for 5 min at 95°C. Samples were run for western blot.

### Alphafold predictions

All predictions were performed using AlphaFold3 via the public server at www.alphafoldserver.com [48]. AlphaFold inputs: BRIP1:BRCA1 interaction—one copy of residues 1640–1863 (BRCT domains) of human BRCA1 (UniProt ID: P38398, RefSeq: NP_009225), and one copy of human BRIP1/FANCJ (UniProt ID: Q9BX63), modified with phosphorylation at Ser990. CtIP:BRCA1 interaction—one copy of BRCT domains, and one copy of human CtIP (UniProt ID: Q99708), modified with phosphorylation at Ser327. ABRAXAS1:BRCA1 interaction–double phosphorylation of ABRAXAS1 at Ser404 and Ser406 induces a 2:2 tetrameric complex between ABRAXAS1 and BRCA1[39]. Additionally, ABRAXAS1 forms an obligate heterodimer with BRCC36 (UniProt ID: P46736) [49]. Therefore, the prediction was performed using two copies each of human ABRAXAS1 (UniProt ID: Q6UWZ7), phosphorylated at Ser404 and Ser406, BRCC36, and BRCT domains. AlphaFold3 correctly and confidently predicted BRCT:ABRAXAS1 dimerization in three out of five models. The remaining two models produced a likely erroneous prediction, in which each BRCT was predicted to bind at the ABRAXAS1:BRCC36 dimer interface, a site that is normally occupied by BABAM2 in the constitutive BRCA1-A core complex [49]. Predictions for BRIP1:BRCA1 and CtIP:BRCA1 did not result in dimer formation, even when a 2:2 stoichiometry was used as input.

All structural figures were rendered using ChimeraX [50].

### Statistical analysis

Statistical analysis of BRCT variant abundance in the Y2H screen was performed using ENRICH2 [47]. All other statistical analyses were conducted in GraphPad Prism version 10.2.3 (GraphPad Software, San Diego, CA). For comparisons between multiple conditions within a group, a one-way mixed-effects ANOVA was performed, assuming Gaussian distribution of residuals. The Geisser–Greenhouse correction was applied to account for violations of sphericity. When comparing multiple conditions to a single control, a one-way ANOVA with Dunnett’s multiple comparisons test was used, with multiplicity-adjusted *P*-values reported for each comparison.

### Artificial intelligence usage

Text editing was supported by ChatGPT and Microsoft Copilot. ChatGPT was additionally used to generate illustrative figures and to assist in developing Python code for visualizing ENRICH2 scores from the Y2H screening data.

## Results

### Setting up a platform to study BRCA1 BRCT domains interactions using yeast-two-hybrid

To investigate how the BRCT domains mediate interaction with its full-length partner proteins, we employed the Y2H method. We fused the Y2H DNA-binding domain and activation domain to either the BRCA1 BRCT domains (amino acids 1646–1859 of the human NP_009225) or the human full-length partner proteins ABRAXAS1, BRIP1, and CtIP. Due to auto-activation observed when CtIP was fused to the DNA-binding domain (Supplementary Fig. S1A), we opted to fuse the BRCT domains to the DNA-binding domain and ABRAXAS1, BRIP1, or CtIP to the activation domain (Fig. 1B). Using a configuration in which both BRCT and partner proteins were N-terminally fused to the Y2H domains, we detected binding between the BRCT domains and ABRAXAS1 and CtIP, as indicated by colony growth on selective medium. However, no binding to BRIP1 was observed (Fig. 1B). Pull-down assays using *in vitro* purified His-tagged BRCT domains with yeast lysates overexpressing BRIP1 showed successful binding (Supplementary Fig. S1B), suggesting that the lack of a detectable interaction via Y2H was not due to improper folding or lack of post-translational modifications of BRIP1 in yeast. Instead, binding probably occurs in an orientation unfavourable for the formation of a functional transcription factor complex. Supporting this, we did observe colony growth when BRIP1 was fused to the C-terminus of the activation domain (Supplementary Fig. S1C), although the growth was too weak for large-scale assays. As a consequence, we focused on ABRAXAS1 and CtIP for the large-scale Y2H screens.

In mammalian cells, the interaction between the BRCT domains and ABRAXAS1 or CtIP depends on the phosphorylation of Serine 406 and Serine 327, respectively. To determine whether this is recapitulated in yeast, we introduced serine to alanine mutations at these residues. As expected, no colony formation was observed on selective plates, indicating a lack of binding between BRCT domains and the ABRAXAS1 S406A or CtIP S327A mutants (Fig. 1C). We also tested the ABRAXAS1 S404A variant that has been shown to affect BRCA1 dimerization, but not BRCA1 binding to ABRAXAS1 [39]. Consistent with the previous report [39], this ABRAXAS1 variant retained binding to the BRCT domains (Fig. 1C). These findings show that the protein interactions in yeast closely resemble those seen in mammalian cells. This supports the use of yeast as a reliable model for large-scale studies of BRCT domain interactions.

### Y2H screens yield amino acid resolution of the interaction between BRCT domains with ABRAXAS1 and CtIP

To characterize the interaction between the BRCT domains and ABRAXAS1 and CtIP at single amino acid scale, we constructed an amino acid-based site-saturated mutagenesis library of the BRCT domains, mutating each residue to all 19 alternative amino acids. This resulted in an oligonucleotide pool of 4,066 unique BRCT variants, and a WT BRCT sequence as control (see the ‘Materials and methods’ section for details on the used library). The oligonucleotide pool was cloned into a plasmid library in-frame with the Y2H DNA-binding domain. Subsequent PCR amplification of the BRCT-encoding region and next-generation sequencing confirmed the successful incorporation and uniform representation of all variants (Supplementary Fig. S2A).

The plasmid library was subsequently transformed into yeast strains expressing either ABRAXAS1 or CtIP fused to the Y2H activation domain and plated on media with or without selection pressure (Fig. 1D). Pooled colonies were harvested from each condition and next-generation sequencing verified that all variants are present on the non-selective plates with uniformity comparable to the original plasmid pool (Supplementary Fig. S2B and C).

**Figure 2.**
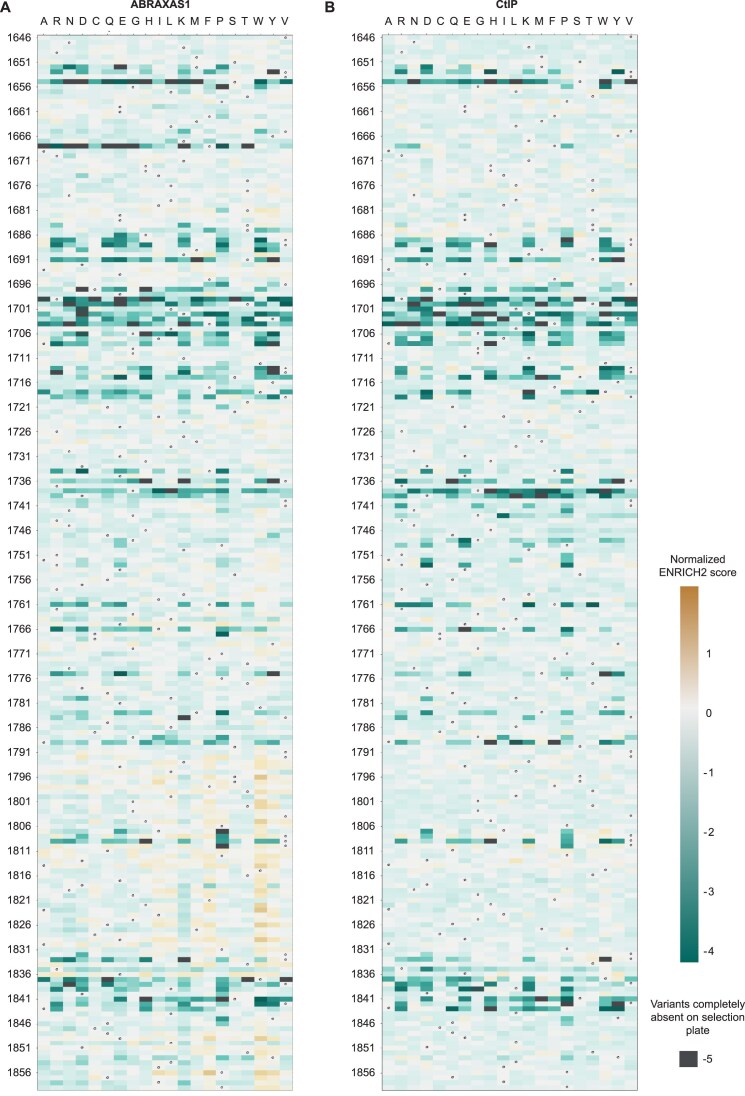
Mapping binding of the BRCT domains with ABRAXAS1 and CtIP at amino acid resolution. (**A**) Heatmap of normalized ENRICH2 scores depicting the effect of all possible amino acid substitutions in the BRCA1 BRCT domains on ABRAXAS1 binding. (**B**) Heatmap of normalized ENRICH2 scores showing the effect of all possible amino acid substitutions in the BRCA1 BRCT domains on CtIP binding. In both panels, the*y*-axis represents BRCA1 residue numbers (human NP_009225, aa 1646–1859), while the*x*-axis denotes the amino acid substitutions. The WT amino acid at each position is marked by a circle in each row. The more negative the score, the more disruptive the mutation; a mutation completely absent in the selection plate (suggestive of complete disruption of binding) is assigned a score of −5, indicated by dark grey shading in the heatmap.

The effect of each mutation on binding was quantified by comparing the relative abundance of each variant between the selective and non-selective plates. This analysis was performed using the ENRICH2 algorithm [47]. The resulting heatmap highlights specific regions within the BRCT domains that are critical for interactions with ABRAXAS1 or CtIP (Fig. 2A and B, and Supplementary Table S2). Notably, BRCA1 residues 1655, 1699, 1701, 1702, 1704, 1775, and 1839, previously identified as key for BRCA1 binding to ABRAXAS1, CtIP, and BRIP1 [34–39], were among the most critical sites for binding in our Y2H setup.

**Figure 3.**
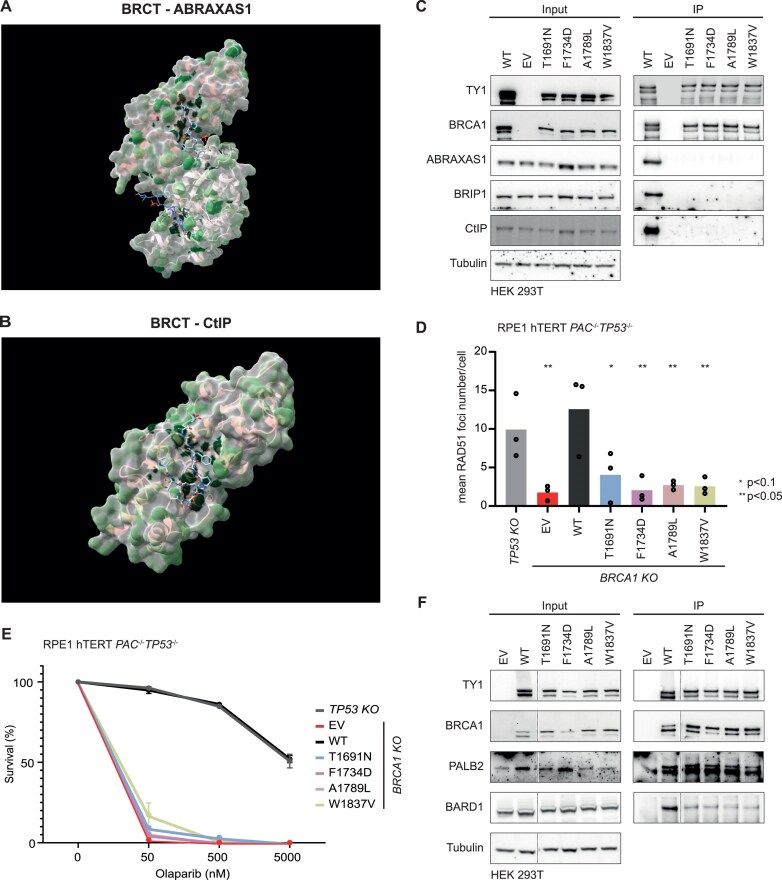
Identification of novel interaction modules between the BRCT domains and ABRAXAS1 and CtIP. (**A**) Superimposition of Y2H data onto the solved crystal structure of the BRCT domains bound to the ABRAXAS1 peptide (PDB: 4Y18). (**B**) Superimposition of Y2H data onto the solved crystal structure of the BRCT domains bound to the CtIP peptide (PDB: 1Y98). For both panels (A) and (B), the score for each residue was calculated by summing the ENRICH2 scores of the individual amino acid substitutions that negatively affected binding. Darker green colouration indicates residues whose mutation led to more severe disruption of binding. (**C**) IP assay using TY1 antibody in HEK 293T cells overexpressing WT or mutant BRCA1-TY1 to assess its interaction with ABRAXAS1, BRIP1, and CtIP at 3 h post-irradiation with 5 Gy. Tubulin serves as a loading control (EV: empty vector). (**D**) Quantification of RAD51 foci in indicated RPE1 hTERT cells virally complemented with EV, BRCA1 WT, or variants by immunofluorescence (IF) at 3 h post-irradiation with 5 Gy for different BRCA1 variants (*n* = 3, representing mean ± SEM). *P-*value was calculated using ANOVA, followed by multiple comparison Dunnett test comparing the mean values to *TP53* KO. Note: *TP53* KO is expressing endogenously tagged BRCA1-TY1. (**E**) Clonogenic survival assay in RPE1 hTERT cells comparing the sensitivity of different BRCA1 variants to increasing concentrations of olaparib (*n* = 3, representing mean ± SEM). Note: *TP53* KO is expressing endogenously tagged BRCA1-TY1. (**F**) IP assay using TY1 antibody in HEK 293T cells overexpressing WT or mutant BRCA1-TY1, showing interactions with BARD1 and PALB2 at 3 h post-irradiation with 5 Gy. Tubulin serves as a loading control (EV: empty vector). Dotted line indicates exclusion of lanes; the compiled lanes originate from the same blot.

To visualize the location of functionally important residues, we mapped our results onto the crystal structures of the BRCT domains in complex with minimal peptides from ABRAXAS1 and CtIP (PDB 4Y18 [39] and 1Y98 [37], respectively; Fig. 3A and B). We observed strong concordance between our functional data and the structural information, with residues directly involved in peptide binding showing high intolerance to mutation. This underscores their essential role in mediating the interaction. These findings demonstrate the robustness of our interaction assays in identifying critical BRCT residues required for binding to ABRAXAS1 and CtIP.

### Y2H data expand insights into structural and binding analysis relevant to human BRCA1

Interestingly, our analysis revealed several novel interaction sites that disrupt interactions between BRCT domains and the partner proteins beyond the previously described areas. To demonstrate that our Y2H approach correctly predicts interactions in human cells, we selected four residues that are highly susceptible to perturbation—T1691, F1734, A1789, and W1837. Changing these amino acids to most other residues disrupted binding to both ABRAXAS1 and CtIP in our Y2H assay (Fig. 2A and B). To validate the Y2H data in a mammalian setting, we chose the following amino acid substitutions for further follow-up: T1691N, F1734D, A1789L, and W1837V. We transiently overexpressed the mutants in the context of full-length TY1-tagged BRCA1 in HEK293T cells and assessed protein–protein interactions via IP assays. Consistent with the Y2H data, IP assays confirmed a loss of interaction for all four mutants with both proteins, as well as BRIP1 (Fig. 3C). Moreover, these mutations were associated with defects in HR in RPE1 hTERT cells, as demonstrated by reduced RAD51 foci formation and increased sensitivity to olaparib (Fig. 3D and E, and Supplementary Fig. S3A). Collectively, these findings establish a correlation between impaired protein binding and HR deficiency, highlighting the functional significance of these residues in BRCA1-mediated DNA repair.

Analysing these specific residues in the described crystal structures showed that A1789, W1837, and F1734 are part of the hydrophobic core (Supplementary Fig. S3B), where mutations could alter folding. Similarly, T1691 is positioned in a loop, and substitutions may affect its packing (Supplementary Fig. S3B). Structural disruptions of these features may therefore explain the loss of binding to partner proteins ABRAXAS1, BRIP1, and CtIP, as well as the observed defect in HR. To explore whether these mutations in the BRCT domains affect the overall structure and/or the interactome of BRCA1, we examined their impact on the binding to canonical partners of other BRCA1 domains. For this, we tested interactions with BARD1, which binds to BRCA1 through the N-terminal RING domain, and PALB2, which binds via the coiled-coil domain (Fig. 1A). Our IP results showed reduced binding to BARD1, while the interaction with PALB2 seemed unaffected (Fig. 3F). These findings suggest that, despite being located further apart in the primary protein sequence, the mutations may induce more substantial disruptions in the RING domain than in the coiled-coil domain. This is an interesting observation, as it suggests potential communication between the BRCT and RING domains, either through direct structural interactions or via indirect mechanisms.

In summary, we identified novel residues within the BRCT domains that mediate its interactions with ABRAXAS1, BRIP1, and CtIP, and concomitantly show HR defects when mutated. Although our data cannot distinguish between lost binding and folding issues, the strong concordance between Y2H data and IP results in mammalian cells demonstrates the potential of the Y2H data to accurately capture key aspects of BRCT folding and binding in a mammalian context.

### Y2H data reveal separation-of-function mutants

**Figure 4.**
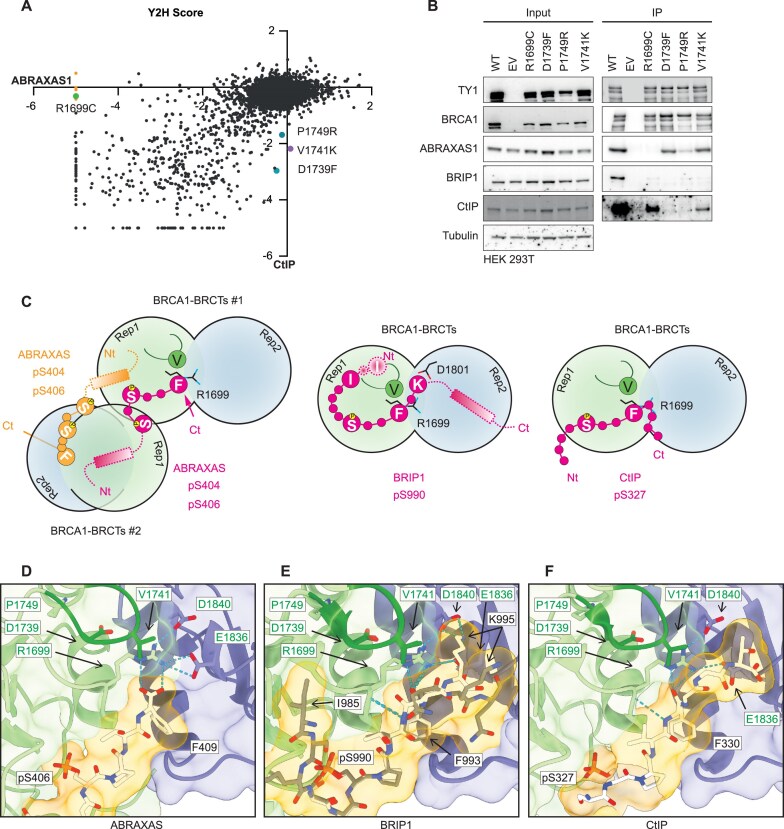
Structural mapping of BRCT separation-of-function mutations. (**A**) Scatter plot correlating ABRAXAS1 Y2H binding scores with CtIP Y2H binding scores. A mutation completely absent in the selection plate is assigned a score of −5. Seperation-of-function mutants selected for further analysis are highlighted as coloured circles. F1668 mutants are highlighted in orange. (**B**) IP assay using TY1 antibody in HEK 293T cells overexpressing WT or mutant BRCA1-TY1, showing interactions with ABRAXAS1, BRIP1, and CtIP at 3 h post-irradiation with 5 Gy X-rays. Tubulin serves as a loading control (EV: empty vector). (**C**) ABRAXAS1, BRIP1, and CtIP bind differently outside of the sPxxF binding pocket. Schematic representation of the structures. ABRAXAS1/BRIP1/CtIP residues resolved in the crystal structure are depicted as pink/orange circles, with key residues labelled in one-letter notation. The direction of the N-terminus (Nt) and C-terminus (Ct) is indicated. The approximate positions of the BRCA1 V1741 loop (green) and BRCA1-V1741 (green circle) are also marked. Interactions predicted by Alphafold (Supplementary Fig. S4D–F) are marked with a dashed line, with helices represented as rectangles. (**D**) ABRAXAS1 binds the BRCTs through its C-terminus. Close-up of the interface between ABRAXAS1 (white) and the BRCTs (BRCT repeat1: green; repeat 2: blue; V1741 loop: dark green). BRCT residues are labelled in green, partner protein residues in black. Hydrogen bonds are shown as dashed lines. Accession code: ABRAXAS1: 4Y18. (**E**) BRIP1 binding extends to BRCT repeat 2. Close-up of the binding interface between BRIP1 and the BRCT domains. BRCT colours as in 4D; BRIP peptide: PDB 1T15, white; PDB 1T29, grey. In 1T29 an interaction is observed between I985 and the backside of the V1741 loop, but K995 does not interact with D1840. (**F**) CtIP extends into the solvent after the F residue. Close-up of the binding interface between CtIP (white) and the BRCT. Residues after F330 extend towards the viewer. BRCT colours as in 4D. Accession codes: CtIP: 1Y98.

In addition to mutations that disrupt binding to all partner proteins, we identified several mutations that selectively impair individual interactions. Most substitutions at residue F1668 predominantly disrupted binding to ABRAXAS1, while having a minimal effect on the CtIP interaction (Fig. 2A). Furthermore, we observed specific amino acid changes in other residues that differentially affected partner binding. These include R1699C, which impaired interaction with ABRAXAS1 but not CtIP, and D1739F, V1741K, and P1749R, which selectively disrupted binding to CtIP but not ABRAXAS1 (Figs 2A and 4A). To validate these Y2H findings, we overexpressed the corresponding BRCA1 mutants in HEK293T cells and assessed their interactions with individual partners using IP (Fig. 4B).

Structural analysis has shown that doubly phosphorylated ABRAXAS1 (S404/S406) induces BRCT domain dimerization [39], whereas BRIP1 and CtIP bind as monomers [36–38] (Supplementary Fig. S4A). Residue F1668 lies immediately adjacent to the BRCT dimerization interface, providing a plausible explanation for why mutations at this position strongly affect ABRAXAS1 binding but have minimal effect on CtIP interaction (Supplementary Fig. S4A). However, this selective disruption was not recapitulated in mammalian IP (Supplementary Fig. S4B).

For the remaining mutations, the interaction profiles observed in the Y2H screen were largely reproduced in the mammalian IP assays. The R1699C mutant exhibited a selective loss of binding to ABRAXAS1 while maintaining its interaction with CtIP (Fig. 4B). Notably, the IP assays also enabled us to evaluate BRIP1 binding, revealing that R1699C disrupts BRIP1 interaction as well (Fig. 4B). The D1739F and P1749R mutants lost binding to CtIP and BRIP1 but preserved interaction with ABRAXAS1 (Fig. 4B), further corroborating the Y2H results. The V1741K mutation does not completely lose its interaction with CtIP, showing slight deviation to the Y2H assay. However, since it specifically lost interaction with BRIP1 while maintaining at least partial binding to both CtIP and ABRAXAS1 (Fig. 4B), V1741K is still a compelling separation-of-function mutant for further investigation. Notably, all four mutants maintained interactions with BARD1 and PALB2, indicating that they do not induce global protein misfolding (Supplementary Fig. S4C)

Structural mapping of our mutants to the known structures and to Alphafold3 predictions provides insight into the basis of this differential binding. While ABRAXAS1, BRIP1, and CtIP all utilize a pS-x-x-F motif to engage the BRCT domain, they diverge structurally beyond this core sequence (Fig. 4C and Supplementary Fig. S4D–F). In ABRAXAS1, the phenylalanine residue constitutes the C-terminal end of the peptide, whereas both BRIP1 and CtIP possess extended C-terminal regions. These extensions result in distinct binding configurations: BRIP1 continues to interact with BRCT repeat 2, while CtIP’s tail projects into the solvent. Consequently, each partner engages the surrounding residues of the phenylalanine-binding pocket—particularly R1699 and V1741—in a unique manner (Fig. 4C–F and Supplementary Fig. S4D–F).

Mutation R1699 is particularly interesting, as it has been widely described as pathogenic, with R1699W and R1699Q known to disrupt binding to all three protein partners [51]. Structurally, the side chain of R1699 forms hydrogen bonds with BRCA1-D1836 and BRCA1-D1840 (Fig. 4D–F), playing a critical role in stabilizing the interface between the two BRCT repeats within the same BRCA1 molecule. Mutation R1699C likely affects binding of ABRAXAS1 by removing two hydrogen bonds formed by the C-terminus and introducing a potential charge repulsion between ABRAXAS1 and the cysteine thiol (–SH) (Fig. 4D). For BRIP1, the same mutation eliminates the hydrogen bond with the backbone of BRIP1-F993 and might potentially affect the positioning of BRCA1-D1840 and its hydrogen bonding to BRIP1 (Fig. 4E). By contrast, CtIP binding is less affected: R1699C disrupts only a single hydrogen bond with CtIP-F330 (Fig. 4F). Together, these observations suggest that CtIP incurs a smaller energetic penalty from the loss of the R1699 side chain compared to ABRAXAS1 and BRIP1, providing a structural rationale for the differential effects of this mutation on partner binding, as observed in both Y2H and IP assays.

The V1741K mutation does not impair binding to ABRAXAS1 or CtIP, as neither protein makes substantial contact with this residue (Fig. 4D and F). However, substitution to the bulky, positively charged lysine side chain is predicted to sterically and electrostatically clash with BRIP1 residues Q994 and K995, thereby disrupting the interaction (Fig. 4E).

Mutations D1739F and P1749R cluster at the base of the loop that bears V1741. These mutations are likely to induce localized misfolding of that loop and misplace V1741. Indeed, D1739Y, D1739V, D1739E, D1739G, and P1749R have been shown to affect BRCT proteolytic stability and overall function [52]. Because ABRAXAS1 has very little interaction with V1741, its binding is almost unaffected by these mutations (Fig. 4D). In contrast, BRIP1 interacts extensively with V1741 through the two residues following the pSxxF motif, likely leading to a loss of binding energy (Fig. 4E). Additionally, I985 of BRIP1 has specific interactions with the backside of the loop that might be affected (Fig. 4E). The mechanism underlying CtIP’s reduced binding, despite limited interaction with the V1741 loop, remains unclear.

In summary, we identified four mutations—R1699C, D1739F, V1741K, and P1749R—that act as separation-of-function variants. These findings offer new avenues to investigate BRCA1’s partner-specific roles, particularly in the context of HR.

### Loss of interaction with BRCA1 partner proteins causes variable defects in homologous recombination

**Figure 5.**
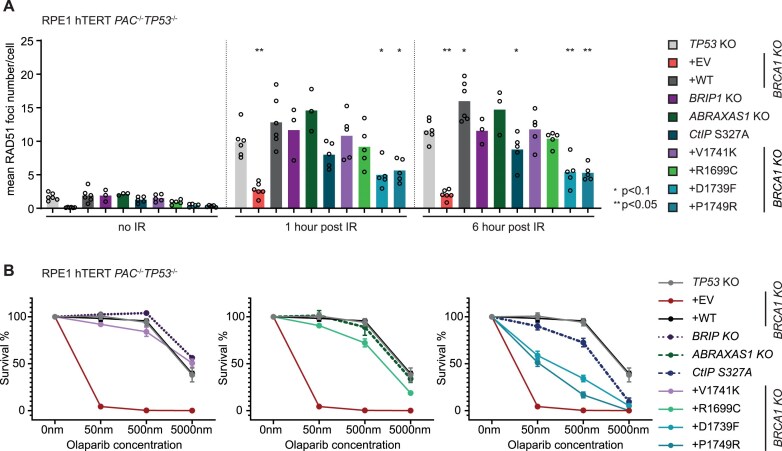
Functional consequences of BRCT separation-of-function mutations. (**A**) Quantification of RAD51 foci observed by IF at 1 h and 6 h post-irradiation with 5 Gy for cells expressing different BRCA1 variants and interaction partner mutants. Foci numbers were analysed using ImageJ macros (*n* = 3–6). *P-*value calculated using ANOVA followed by multiple comparison Dunnett test comparing the mean values to *TP53* KO. Note:*TP53* KO is expressing endogenously tagged BRCA1-TY1. EV: empty vector. (**B**) Clonogenic survival assay comparing the sensitivity of cells expressing different BRCA1 variants and interaction partner mutants to increasing concentrations of olaparib (*n* = 3–9, representing mean ± SEM). Note:*TP53* KO is expressing endogenously tagged BRCA1-TY1. EV: empty vector.

To evaluate the phenotypic effects of the above-described mutations constituting differential loss of protein partners, we assessed RAD51 foci formation as a readout of HR efficiency. We measured RAD51 foci formation in RPE1 cells at 1 and 6 h post-irradiation. First, we examined the impact of partner protein loss by either knocking out (KO) ABRAXAS1 and BRIP1 or introducing an endogenous S327A mutation in CtIP, as cell lines with complete knockout of CtIP are inviable. Consistent with previous studies [29, 53], ABRAXAS1 or BRIP1 loss did not significantly impair RAD51 foci formation, with ABRAXAS1 KO showing a slight but non-significant increase. In contrast, the CtIP S327A mutation caused a moderate decrease in RAD51 foci, confirming its importance for HR (Fig. 5A and Supplementary Fig. S5A).

Next, we assessed the effects of BRCA1 separation-of-function mutants, by comparing their effect to the loss of the respective partner proteins. The V1741K mutant, which specifically disrupts BRIP1 binding, did not affect RAD51 foci formation, phenocopying direct loss of BRIP1. The R1699C mutant, which disrupts interactions with both ABRAXAS1 and BRIP1, showed a modest but non-significant decrease in RAD51 foci, displaying more pronounced effects than losing ABRAXAS1 or BRIP1 alone. The D1739F and P1749R mutants, which impair interactions with both CtIP and BRIP1, caused a significant reduction in RAD51 foci, greater than that observed with the CtIP S327A mutation or BRIP1 KO individually. These findings suggest that simultaneous loss of multiple partner protein interactions exacerbates defects in RAD51 foci formation (Fig. 5A).

We further evaluated HR by assessing the sensitivity of mutants to the PARP inhibitor olaparib. Consistent with the RAD51 foci data, the loss of individual interactions, as observed in ABRAXAS1 KO, BRIP1 KO, and BRCA1 V1741K mutants, did not result in a significantly increased sensitivity to olaparib. In contrast, the loss of binding to CtIP, as well as the combined loss of ABRAXAS1 or CtIP together with BRIP1 led to a more pronounced sensitivity to olaparib, as observed in CtIP S327A, BRCA1 R1699C, D1739F, and P1749R mutants (Fig. 5B).

These findings suggest that the lost interaction with multiple partner proteins has a synergistic effect on HR. Alternatively, the selected BRCA1 mutations may disrupt interactions with other BRCA1-binding partners not examined in this study or impair additional BRCA1 functions, collectively contributing to the observed HR defects.

### BRCT domains interaction data can predict BRCA1 functionality and clinical classification of BRCT mutations

Previously, the loss of HR function in BRCA1 mutants has been used as a key criterion for determining the pathogenicity of BRCA1 variants. Given the HR effects we observe when BRCT interactions are disturbed, we were prompted to investigate whether BRCA1 mutations that disrupt binding to ABRAXAS1 and CtIP consistently correlate with BRCA1 loss of function.

**Figure 6.**
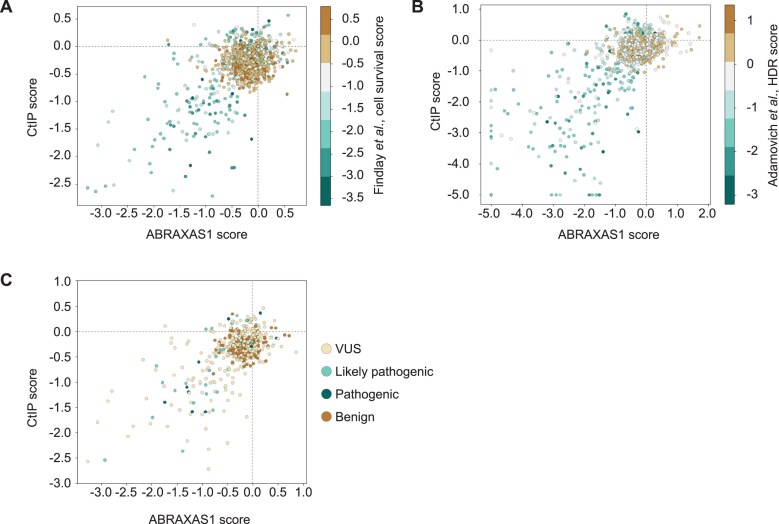
Y2H data show strong correlation with BRCA1 functionality. (**A**) Scatter plot of ABRAXAS1 Y2H binding scores versus CtIP Y2H binding scores. A mutation completely absent in the selection plate is assigned a score of −5. Data points are colour-coded based on functionality scores from Findlay *et al.* [41]. (**B**) Scatter plot of ABRAXAS1 Y2H binding scores versus CtIP Y2H binding scores. A mutation completely absent in the selection plate is assigned a score of −5. Data points are colour-coded based on HDR scores from Adamovich *et al.*[42]. (**C**) Scatter plot of ABRAXAS1 Y2H binding scores versus CtIP Y2H binding scores. A mutation completely absent in the selection plate is assigned a score of −5. Data points are colour-coded according to their ClinVar classification.

To explore this, we correlated our Y2H binding data with established datasets, including those from Findlay *et al.* [41] and Adamovich *et al.* [42] linking large BRCA1 mutational libraries to cellular survival or HR-functionality using GFP-based reporter assays, respectively. Our analysis revealed a strong correlation between mutations that disrupt interactions with ABRAXAS1 and CtIP and these previously reported datasets (Fig. 6A and B, and Supplementary Table S3).

We further compared our findings with the ClinVar classification of BRCA1 mutations. Remarkably, mutations classified as benign in ClinVar consistently retained interactions with ABRAXAS1 and CtIP in our assays, while the majority of mutations classified as pathogenic or likely pathogenic exhibited disrupted binding (Fig. 6C and Supplementary Table S3). Furthermore, the ClinVar database includes numerous variants of uncertain significance (VUS), some of which, according to our Y2H data, also display a loss of interaction with ABRAXAS1 and CtIP. Given the clear functional and clinical correlations observed with other mutants, our Y2H binding scores may provide a valuable framework for reclassifying certain VUS mutations, offering new insights into their potential pathogenicity.

Overall, our findings reveal a clear correlation between the loss of BRCA1 interactions with its protein partners and the impairment of HR or cell survival. Notably, we also identified mutations that, while not disrupting interactions with ABRAXAS1 or CtIP, still result in HR defects, cell viability, and pathogenicity (Fig. 6A–C). This suggests that the role of the BRCT domains in HR extends beyond its interactions with ABRAXAS1 and CtIP, indicating the presence of additional, yet unexplored, mechanisms underlying BRCA1 BRCT domain-mediated HR regulation.

## Discussion

In this study, we performed a large-scale Y2H study to investigate the interactions between the full-length ABRAXAS1 or CtIP proteins and the BRCT domains of BRCA1. Our data confirmed the previously described interaction modes using phospho-peptides of the interactors (Fig. 3A and B) and expanded on them to provide a complete interaction map. We identified previously unknown regions important for interaction and HR (Fig. 3C–E) and found separation-of-function mutations that differentially disrupt interactions, with CtIP binding showing the strongest effect on HR (Figs 4 and 5). Additionally, we observed a correlation between partner protein interaction loss and impaired BRCA1 functionality and pathogenicity (Fig. 6).

Structurally, our Y2H results correlate well with the established crystal structures of the phospho-peptides of ABRAXAS1 or CtIP with BRCA1 as the residues previously identified as critical for binding are also the most significant in our screens (Fig. 3A and B). We also identified additional residue changes—T1691N, F1734D, A1789L, and W1837V—that appear to disrupt interactions with all three partner proteins when mutated. However, we have to note that these effects might stem from general domain destabilization rather than specific interaction defects. The mutation A1789S has been described previously to be protease resistant, while other amino acid changes of the other residues (T1691K, T1691I, F1734S, W1837R, W1837G, W1837C) have been described to be thermal or protease sensitive, indicating compromised folding [52, 54]. Our IP data suggest this instability further, which showed that these mutations disrupt BRCA1’s interaction with BARD1 while having a lesser effect on PALB2 binding (Fig. 3F). Based on these data, it seems that mutations in the BRCT domains can influence the RING domain more significantly than the coiled-coil domain, despite being further apart in the linear protein sequence. This suggests a possible connection between the BRCT domains and the RING domain, either through structural coupling or an indirect effect through the overall interactome. Such interdependence may contribute to the pathogenicity of BRCT mutations beyond their immediate impact on the direct partner protein interactions.

Our data support earlier structural studies by confirming that ABRAXAS1 and CtIP primarily interact with BRCA1 through the established phospho-binding pocket within the BRCT domains [37, 39]. Despite this common anchoring site, the binding modes differ. ABRAXAS1 binds via its C-terminal end, resulting in a shorter interaction surface than that of CtIP or BRIP1. In contrast, both CtIP and BRIP1 have extended C-terminal regions. These extensions lead to distinct binding configurations: BRIP1 maintains contact with BRCT repeat 2, while CtIP’s tail extends outward into the solvent. As a result, each protein engages residues surrounding the phenylalanine-binding pocket—especially R1699 and V1741—in a partner-specific manner (Fig. 4), revealing multiple separation-of-function mutants.

These separation-of-function mutants allowed us to confirm that the BRCA1–CtIP interaction is crucial for HR (Fig. 5A and B). Notably, the combined loss of interactions with two partner proteins, such as ABRAXAS1 and BRIP1 or CtIP and BRIP1, resulted in a more severe impact on HR than the loss of either partner alone. Alternatively, this observation could suggest the existence of additional BRCT domain-interacting partners that are important for HR. Indeed, numerous other proteins such as Aurora A, MEKK3, and UHRF1 are known to bind through the BRCT domains [55–57]. Therefore, the disruption of interactions with these proteins, or with others yet to be discovered, might contribute to the further decrease in HR efficiency or increased sensitivity to olaparib observed in these separation-of-function mutants.

The V1741K mutant of BRCA1 selectively disrupts its interaction with BRIP1 while retaining interactions with ABRAXAS1 and CtIP (Fig. 4B). This specificity makes the V1741K mutant a valuable tool for studying the functional role of the BRCA1–BRIP1 interaction. Previous studies have primarily investigated this interaction using BRIP1 knockout models or mutations such as BRIP1 S990A or BRCA1 R1699, which disrupt interactions with multiple partner proteins. These studies led to conflicting reports regarding BRIP1’s role in HR [9, 23, 31–33]. Our use of the interaction-specific V1741K mutant allowed us to isolate the effect of losing BRIP1 binding. We observed that disruption of the BRCA1–BRIP1 interaction alone does not impair HR. This mutant can thus serve as a more precise tool for exploring the role of the BRCA1–BRIP1 complex, in HR, or in other cellular processes.

The comparative analysis between our structural mapping and published BRCA1 mutation data reveals a clear correlation between the loss of partner protein interactions, impaired HR, and BRCA1 pathogenicity (Fig. 6). This insight serves two significant purposes. First, for mutations already classified as pathogenic, our data provide evidence to determine whether their pathogenicity is due to disrupted interactions with partner proteins. Second, for VUS that clearly disrupt BRCT domain interactions and hence likely impair BRCA1 function, our findings can support their reclassification as pathogenic. This dual application of our data enhances the clinical interpretation of BRCA1 variants, contributing to more accurate genetic counselling and improved cancer risk assessment.

Finally, we identified mutations that, despite retaining interactions with ABRAXAS1 and CtIP, still impair HR and overall BRCA1 functionality (Fig. 6). These findings highlight the importance of the BRCT domains beyond their well-established role as a binding platform for ABRAXAS1, BRIP1, and CtIP. It seems that these tandem domains may also have additional, yet-to-be-discovered functions that contribute to HR, and therefore genome stability. Future studies should investigate these potential non-canonical roles to further elucidate BRCA1’s multifaceted contributions to tumour suppression.

## Supplementary Material

gkaf848_Supplemental_Files

## Data Availability

All raw data, including uncropped western blots, Y2H sequencing results, examples of clonogenic survival and yeast plates, chimeraX scripts for superimposing Y2H data on available BRCT structures, and numerical data are available in Mendeley Data, at https://doi-org/10.17632/9fckzvn6wf.1.

## Notes

Present address: Institute of Molecular Cancer Research, University of Zurich, Strickhofstrasse 40a, CH-8057 Zurich, Switzerland
