# Supplementary material for: Profiling BRCA1-BRCT interactions and their functional relevance at amino acid resolution
Source: Nucleic Acids Res. 2025 Sep 17;53(17):gkaf848. doi: 10.1093/nar/gkaf848 (PMC12448885; doi:10.1093/nar/gkaf848)
Supplement: gkaf848_Supplemental_Files [file gkaf848_supplemental_files.zip › Supplementary_data.pdf]

Profiling BRCA1-BRCT interactions and their functional relevance at amino acid-resolution

Venda Mangkusaputra[1,2], Andrea G. Murachelli[2,3], Zhengzhou Yu[1], Annouche den Hollander[1], Roberta Menafra[4], Anne Schreuder[1,2,5], Susan L. Kloet[4], Titia K. Sixma[2,3], Sylvie M. Noordermeer[1,2,*]

Supplementary Data

- Supplementary table descriptions
- Supplemental figures 1-5

Supplementary table descriptions

Supplementary table 1: Primer sequences and descriptions

Primer sequences used in this study, along with relevant descriptions for each.

Supplementary table 2: Y2H large-scale assay data

Sequencing read counts for each BRCA1 BRCT variant detected in the non-selective ("All") and selective ("Binders") plates from three independent replicates of the yeast two-hybrid (Y2H) assay, testing interactions with ABRAXAS1 (ABX) or CtIP.

The table includes binding scores calculated using the ENRICH2 pipeline, along with associated standard errors and p-values.

Scores for overlapping amino acid regions (3–4 residues) between BRCT fragment 1 (BRCT1) and fragment 2 (BRCT2), or between BRCT2 and fragment 3 (BRCT3), were used to normalize scores across the three BRCT fragments.

Cells marked as #VALUE, N/A, or left empty indicate variants completely depleted in the selective plate ("Binders"), making ENRICH2 score calculation (log ratio) not possible.

The three different BRCT fragments (BRCT 1/2/3) are reported in the different sheets.

Supplementary table 3: Comparison of Y2H scores with functional and clinical databases

Binding scores of BRCA1 variants from the Y2H assay compared to published datasets reporting BRCA1 functional impact or clinical classification.

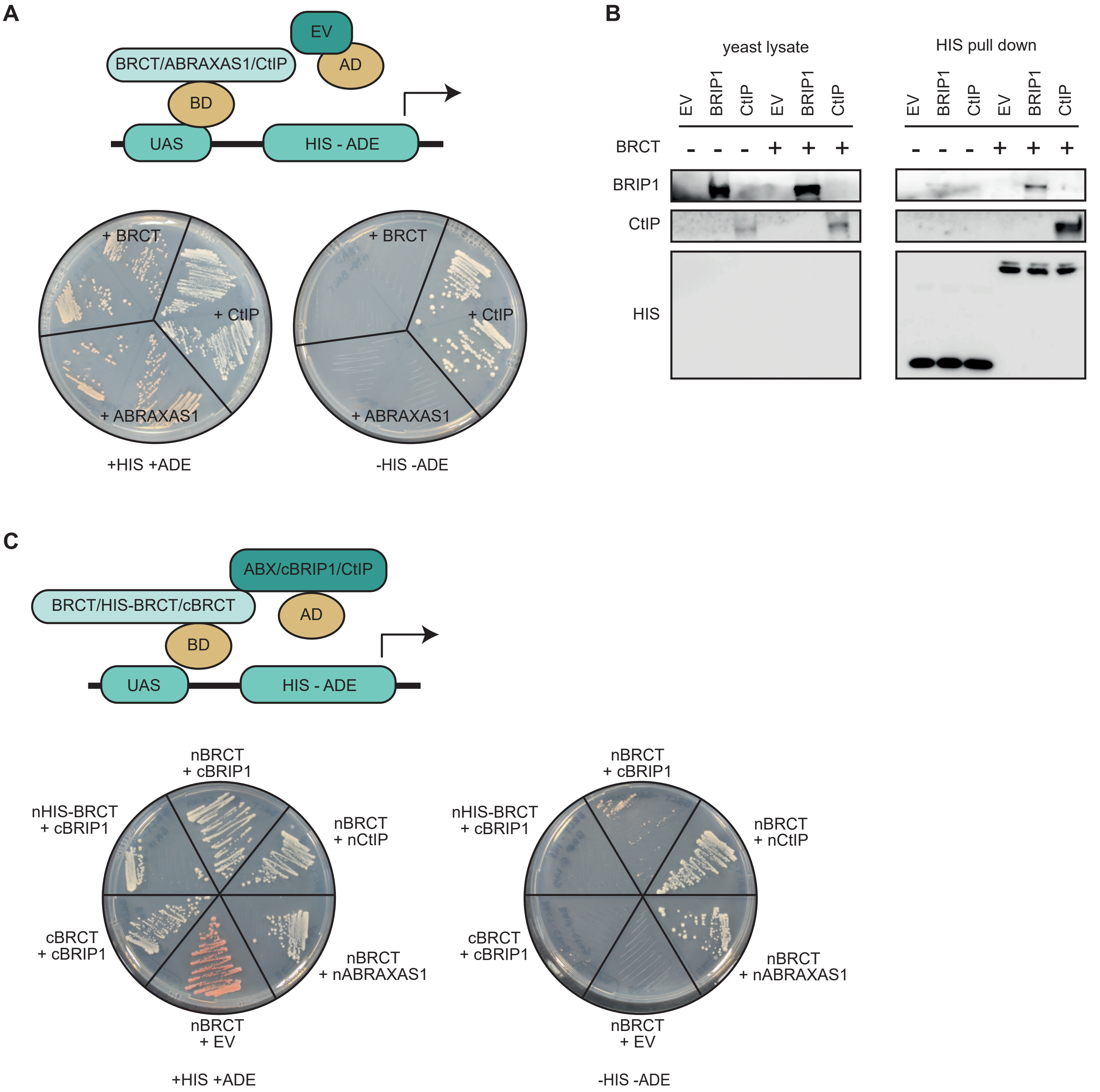

Supplemental Figure 1 (related to figure 1)

(a) Restreaking assay of yeast colonies on non-selective medium (+HIS +ADE) or selective medium (-HIS -ADE), demonstrating autoactivation of CtIP when fused to the DNA-binding domain (BD).

(b) Pull-down assay in yeast extracts overexpressing human CtIP and BRIP1 using nickel beads to capture *in vitro* purified HIS-tagged BRCT, demonstrating interactions between HIS-BRCT and CtIP and BRIP1 (EV: empty vector).

(c) Restreaking assay of yeast colonies on non-selective medium (+HIS +ADE) or selective medium (-HIS -ADE) using N- or C-terminal tagging of protein to Y2H domains (designated nXXX or cXXX, respectively), demonstrating a low-affinity interaction between BRIP1, fused to the C-terminus (cBRIP1) of the Y2H activation domain (AD), and BRCT, fused to the N-terminus of the Y2H DNA-binding domain (BD). (EV: empty vector).

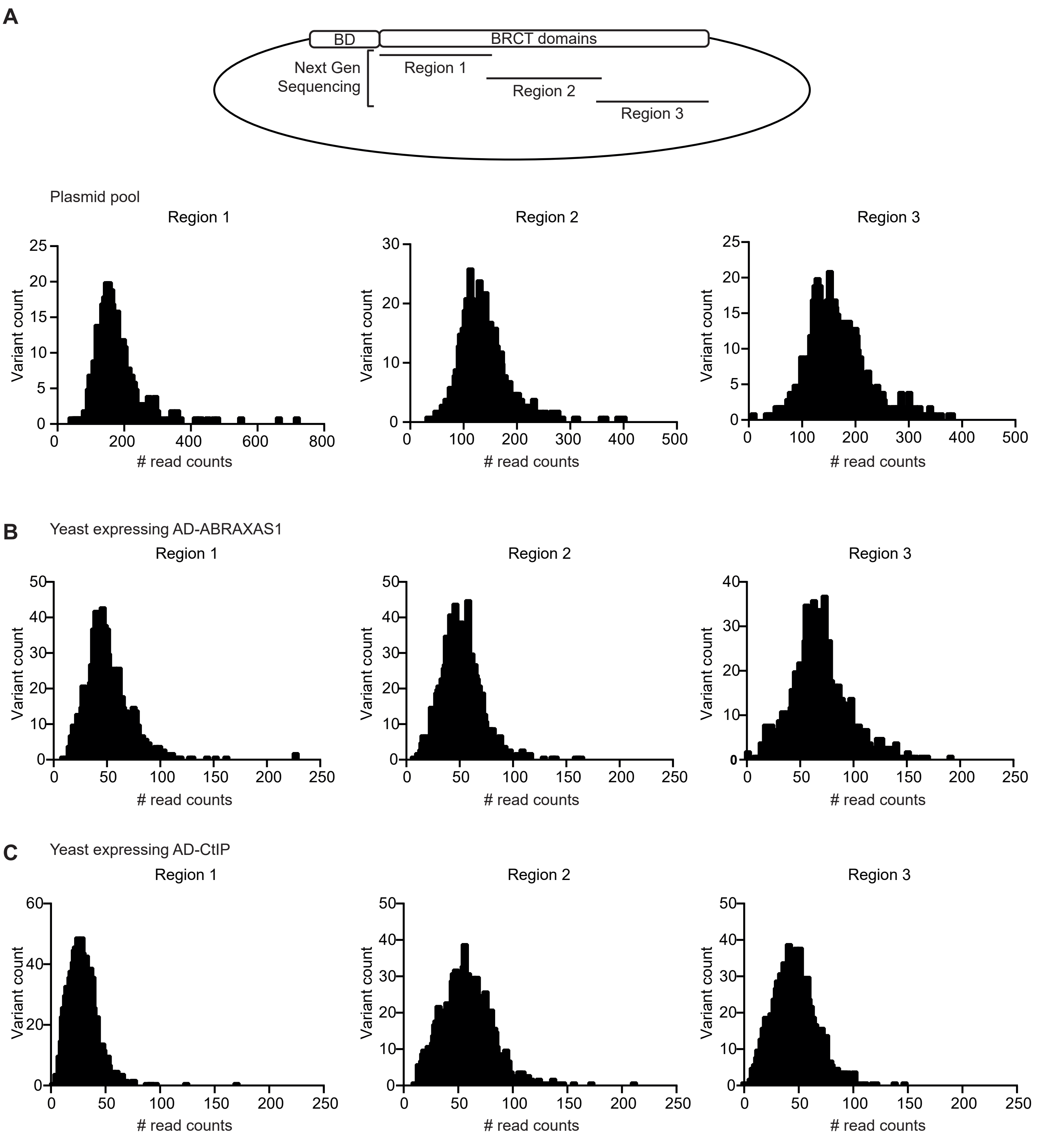

Supplemental Figure 2 (related to figure 2)

(a) Top: next-generation sequencing was used to analyze the abundance of all variants within the BRCT domains. To allow coverage of the full domain, sequencing was done based on 3 subregions, sharing 9-12 bps overlap. Bottom: histogram illustrating the distribution of variant counts across the three different sequenced regions of the BRCT domains following library cloning into Y2H plasmid.

(b) Histogram showing the distribution of variant counts across the three sequenced regions of the BRCT domains after library transformation into yeast expressing AD-ABRAXAS1 in non-selective medium.

(c) Histogram displaying the distribution of variant counts across the three sequenced regions of the BRCT domains after library transformation into yeast expressing AD-CtIP in non-selective medium.

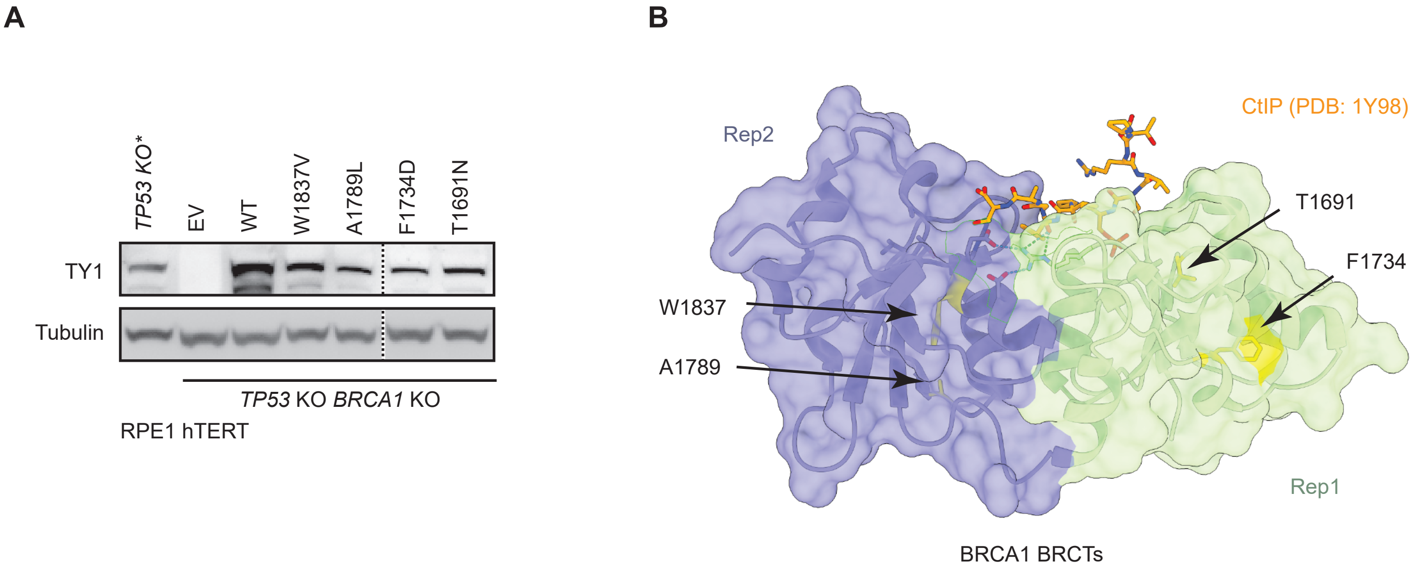

Supplemental Figure 3 (related to figure 3)
(a) Western blot showing the expression level of virally introduced TY1-tagged BRCA1 WT/mutants in a *TP53* KO *BRCA1* KO background compared to the expression of endogenously tagged BRCA1-TY1 in RPE1 hTERT *TP53* KO cells (indicated by *) used in the clonogenic survival and immunofluorescent experiments. Tubulin serves as a loading control (EV: empty vector). Dotted line indicates exclusion of lanes; the compiled lanes originate from the same blot.
(b) A1789, W1837, T1691 and F1734 are buried in the BRCT core. The named residues are shown in yellow within the structure of BRCA1-BRCTs in complex with CtIP (PDB: 1Y98). BRCT repeats one and two are shown in green and blue respectively .

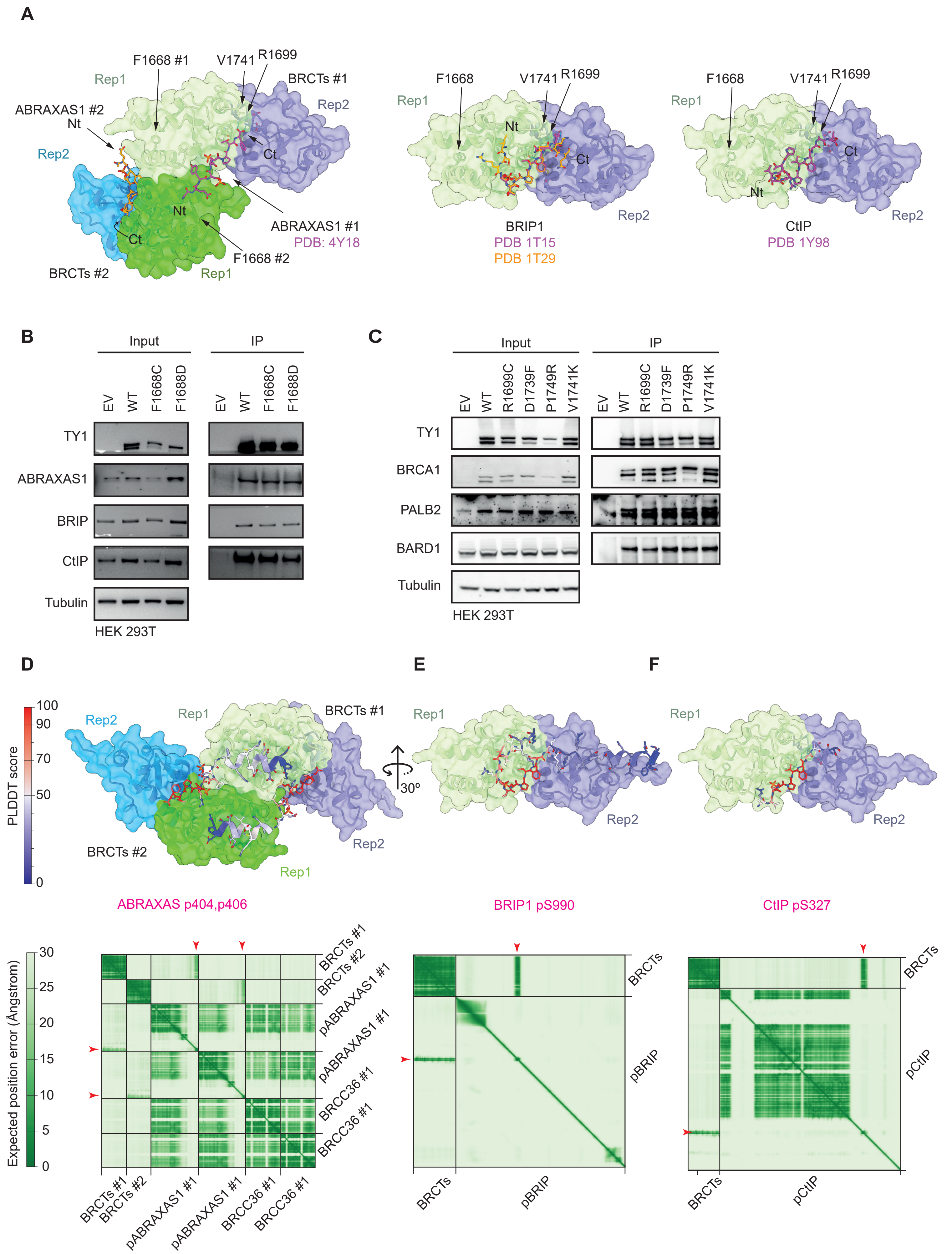
A
F1668 #1
V1741
R1699
BRCTs #1
Rep1
Rep2
ABRAXAS1 #2
Nt
Ct
Rep2
Nt
Ct
ABRAXAS1 #1
PDB: 4Y18
F1668 #2
BRCTs #2
Rep1
F1668
V1741
R1699
Rep1
Nt
Ct
Rep2
BRIP1
PDB 1T15
PDB 1T29
F1668
V1741
R1699
Rep1
Ct
Nt
Rep2
CtIP
PDB 1Y98
B
Input
IP
EV
WT
F1668C
F1688D
TY1
ABRAXAS1
BRIP
CtIP
Tubulin
HEK 293T
C
Input
IP
EV
WT
R1699C
D1739F
P1749R
V1741K
TY1
BRCA1
PALB2
BARD1
Tubulin
HEK 293T
D
E
F
PLDDT score
100
90
70
50
0
Rep2
Rep1
BRCTs #1
BRCTs #2
Rep1
Rep2
30º
Rep1
Rep2
Rep1
Rep2
ABRAXAS p404,p406
BRIP1 pS990
CtIP pS327
Expected position error (Ångstrom)
30
25
20
15
10
5
0
BRCTs #1
BRCTs #2
pABRAXAS1 #1
pABRAXAS1 #1
BRCC36 #1
BRCC36 #1
BRCTs
pBRIP
BRCTs
pCtIP

Supplemental Figure 4 (related to figure 4)

(a) Binding of ABRAXAS1, but not BRIP1 or CtIP induces BRCT dimerization. Crystal structures of peptides of ABRAXAS1 (left), BRIP1 (center) and CtIP (right) bound to BRCT domains. Peptides and BRCTs are shown in stick and surface representation, respectively. The ABRAXAS1 complex shows a 2:2 stoichiometry, with each protomer labelled. BRIP1 and CtIP bind as monomers. Colors: BRCT repeat 1: green; BRCT repeat 2: blue; peptide: orange / purple. Accession codes: ABRAXAS1: 4Y18, BRIP1: 1T15, 1T29, CtIP: 1Y98.

(b) Immunoprecipitation (IP) assay using TY1 antibody in HEK 293T cells overexpressing BRCA1-TY1, demonstrating interactions of different BRCT variants with ABRAXAS, BRIP1, and CtIP at 1 hour post-irradiation with 5 Gy. Tubulin serves as a loading control (EV: empty vector).

(c) IP assay using TY1 antibody in HEK 293T cells overexpressing BRCA1-TY1, showing interactions with BARD1 and PALB2 at 3 hours post-irradiation with 5 Gy. Tubulin serves as a loading control (EV: empty vector).

(d) Alphafold3 prediction of the binding of ABRAXAS1 s404p,s406p to the BRCA1-BRCTs. Top: the ABRAXAS1 complex shows a 2:2 stoichiometry, with each protomer labelled. Alphafold predicts the interactions observed in the crystal structure with high confidence. Additionally, it predicts, consistently but with low confidence, an extra interactions for ABRAXAS1, where the N-terminal residues 386-400 form a helix that interacts with the opposite BRCT domain. The prediction was carried out for a 2:2:2 BRCTS:BRCC36:doubly phosphorylated ABRAXAS1 complex, but only the BRCTs and the ABRAXAS1 peptides are shown. BRCT domains and bound peptides are shown in surface and stick representation, respectively. Colors: peptide: pLDDT coloring; BRCT repeat 1: green; BRCT repeat 2: blue. pLDDT score for the BRCTs is >85. Bottom: predicted alignment error matrix of the prediction. Labels and sectors indicate which portion of the collated sequence corresponds to each protein. Red arrows indicate the positions corresponding to the ABRAXAS1 phosphopeptide.

(e) Alphafold prediction of binding of BRIP1 s990p to BRCA1. Top: Alphafold predicts the interactions observed in the crystal structure with high confidence. Additionally, it predicts, consistently but with low confidence, that the binding of BRIP1 extends C-terminally, with residues 1001-1013 forming an amphipatic helix that interacts with BRCT repeat 2. The prediction was carried out for a 1:1 BRCTs:BRIP complex, but only BRCTs and the BRIP1 peptide are shown. Colors as in Supplementary figure 4d. Bottom: predicted aligned error matrix. Annotations as in Supplementary figure 4d.

(f) Alphafold prediction of binding of CtIP s990p to BRCA1. Top: Alphafold prediction is identical to the crystal structure. The prediction was carried out for a 1:1 BRCTs:CtIP complex, but only the BRCTs and the CtIP peptide are shown. Colors as in Supplementary figure 4d. Bottom: predicted aligned error matrix. Annotations as in Supplementary figure 4d.

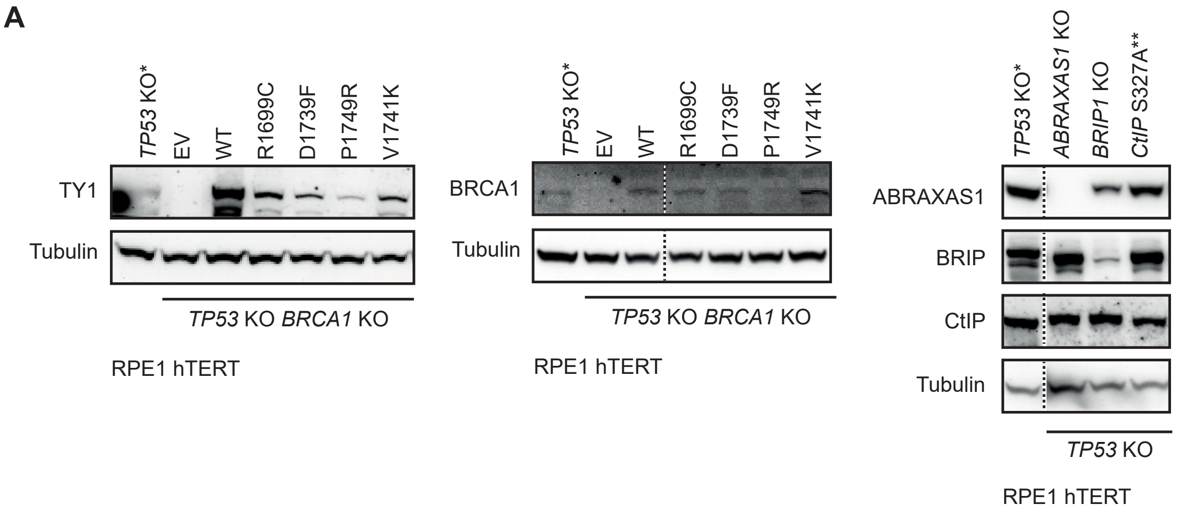

Supplemental Figure 5 (related to figure 5)

(a) Western blot to show the protein expression of the virally transduced cell lines expressing BRCA1-TY1 WT, BRCA1-TY1 mutants, and different BRCA1 partner protein KOs or mutant used for clonogenic survival and immunofluorescent experiments. Tubulin serves as a loading control (EV: empty vector).*The control cells used in these experiments are RPE1 hTERT *TP53* KO cells with a TY1 tag endogenously introduced into the BRCA1 coding sequence. ** These cells carry an endogenously introduced S327A mutation in the *CtIP* coding sequence. Dotted line indicates exclusion of lanes; the compiled lanes originate from the same blot. EV: empty vector.
